# Lightweight SM-YOLOv5 Tomato Fruit Detection Algorithm for Plant Factory

**DOI:** 10.3390/s23063336

**Published:** 2023-03-22

**Authors:** Xinfa Wang, Zhenwei Wu, Meng Jia, Tao Xu, Canlin Pan, Xuebin Qi, Mingfu Zhao

**Affiliations:** 1 School of Information Engineering, Henan Institute of Science and Technology, Xinxiang 453003, China; 2 Faculty of Engineering and Technology, Sumy National Agrarian University, 40000 Sumy, Ukraine; 3 College of Mechanical and Electrical Engineering, Xinxiang University, Xinxiang 453003, China; 4 Institute of Farmland Irrigation, Chinese Academy of Agricultural Sciences, Xinxiang 453002, China

**Keywords:** tomato detection, YOLOv5, small-target detection, lightweight

## Abstract

Due to their rapid development and wide application in modern agriculture, robots, mobile terminals, and intelligent devices have become vital technologies and fundamental research topics for the development of intelligent and precision agriculture. Accurate and efficient target detection technology is required for mobile inspection terminals, picking robots, and intelligent sorting equipment in tomato production and management in plant factories. However, due to the limitations of computer power, storage capacity, and the complexity of the plant factory (PF) environment, the precision of small-target detection for tomatoes in real-world applications is inadequate. Therefore, we propose an improved Small MobileNet YOLOv5 (SM-YOLOv5) detection algorithm and model based on YOLOv5 for target detection by tomato-picking robots in plant factories. Firstly, MobileNetV3-Large was used as the backbone network to make the model structure lightweight and improve its running performance. Secondly, a small-target detection layer was added to improve the accuracy of small-target detection for tomatoes. The constructed PF tomato dataset was used for training. Compared with the YOLOv5 baseline model, the mAP of the improved SM-YOLOv5 model was increased by 1.4%, reaching 98.8%. The model size was only 6.33 MB, which was 42.48% that of YOLOv5, and it required only 7.6 GFLOPs, which was half that required by YOLOv5. The experiment showed that the improved SM-YOLOv5 model had a precision of 97.8% and a recall rate of 96.7%. The model is lightweight and has excellent detection performance, and so it can meet the real-time detection requirements of tomato-picking robots in plant factories.

## 1. Introduction

Plant factories are an innovative vertical agriculture solution, representing an advanced form of greenhouse agriculture capable of producing sustainable supplies of vegetables, herbs, flowers, and other crops throughout the year, based on relatively controlled environmental conditions [1]. They also serve as an urban agriculture solution, providing fresh, nutritious, and high-quality plant products to urban areas, allowing city residents to consume freshly harvested vegetables [2]. Tomatoes are highly valued and widely grown in greenhouses and plant factories. In 2020, the global tomato cultivation area was approximately 5.05 million hectares, with an annual output of 186 million tons [3]. In tomato target detection, the dense foliage of tomato plants frequently obstructs small-target tomato varieties, resulting in a lower detection accuracy. Additionally, to enhance detection accuracy, detection models commonly depend on complex and large heavyweight models, which necessitate a high computing power and storage capacity and escalate the manufacturing costs of mobile and intelligent devices. These limitations hinder the use of robots for pruning, pollination, harvesting, and other operations in plant factories.

In the automated and intelligent production management process of tomato plant factories, in addition to inspection and picking robots, intelligent control platforms also require the monitoring of tomato growth and yield estimation. This requires the ability to recognize and detect tomatoes and accurately determine the position and quantity of tomato fruits. Traditional detection methods are mainly based on shape and color feature extraction, making logical judgments based on the extracted information. Traditional target detection methods include scale-invariant feature transform (SIFT) [4], histogram of oriented gradient (HOG) [5], support vector machine (SVM) [6], and selective search for object recognition [7]. Iwasaki et al. [8] presented a detection approach for mini tomatoes that utilized hue information and candidate area curvature, achieving a detection rate of 78.8%. Linker et al. [9] employed color and smoothness-based detection to estimate the number of green apples in an orchard environment, achieving a high level of correct detection accuracy, albeit at the cost of poor robustness. Wei et al. [10] proposed a method that used an improved Otsu threshold algorithm to extract color features in the Ohta color space [11], followed by segmentation using the Otsu threshold algorithm. The results obtained for detecting the four fruits tested in the experiment were favorable. However, it is worth noting that methods based solely on color features typically have less robustness. Li et al. [12] proposed a detection method for tomato fruit using the fast normalization cross-correlation function (FNCC) and circular Hough transform (CHT) detection methods. They achieved favorable outcomes on the test dataset. However, the algorithm was susceptible to changes in the environment and was applicable in only limited scenarios. Fu et al. [13] employed image processing techniques to recognize and detect ripe kiwifruit. The approach relied on performing numerous color-channel- and color-space-based operations, which made it computationally demanding and less robust. In summary, traditional detection methods are highly difficult to design and promote, especially in the case of an insufficient sample size or number of features, and it is difficult to achieve high precision. In addition, the robustness of detection is not high, so it is still challenging to apply these methods in practical situations.

In recent years, with the successful application of deep convolutional neural networks (DCNNs) in agriculture, computer-vision-based DCNN detection algorithms have provided a new research direction for tomato fruit detection and classification. The DCNN target detection methods can be divided into two categories according to the number of detection stages: (1) Two-stage detection methods first enumerate the candidate frames for the image and then classify and predict the candidate frames. Based on convolutional neural networks (CNNs), regional convolutional neural network (RCNN) [14], Fast RCNN [15], Faster RCNN [16], and so on belong to this type of detection method. Two-stage detection models have a high precision and recall performance. However, their application in real-time detection scenarios is challenging due to their large network size and slow operation speed. (2) Single-stage detection methods directly extract features from the input image and then directly locate and classify the target on the extracted features. The single-shot MultiBox detector (SSD) [17] and You Only Look Once (YOLO) series [18,19,20,21,22,23] belong to this type of detection method. Thanks to their network structure design, single-stage detection models have a fast operation speed that can meet real-time performance requirements, and the accuracy can reach the level of two-stage detection models.

With the further development of computer vision, it has been discovered that deeper networks perform better in machine vision. However, the problem of model degradation arises with the further deepening of the network. He et al. [24] addressed this issue by using residual networks to further enhance the network to 152 layers and alleviate the problem of feature degradation. The MobileNetV3-Large backbone network used in this study also extensively employs residual structures to alleviate model degradation and further extract features. Mnih et al. [25] used attention mechanisms in computer vision to reflect the focus of the human gaze in deep learning models. Based on this, spatial attention mechanisms and channel attention mechanisms have also been successful [26,27]. Howard et al. [28] proposed MobileNetV3, a lightweight backbone network, by introducing an SE channel attention mechanism using depthwise separable convolutional and residual structures. Inspired by this, we propose the SM-YOLOv5 model, which replaces the CSPDarknet53 backbone network with MobileNetV3-Large to reduce the model size and maintain high accuracy in the detection of small tomato targets in plant factories.

Zhang et al. [29] used the improved YOLOV4 model for apple fruit detection, implemented the GhostNet feature extraction network with the coordinate attention module in YOLOv4, and introduced depthwise convolution to reconstruct the neck and YOLO head structure, achieving an mAP of up to 95.72%; however, the network scale was large. Xu et al. [30] used the improved YOLOv5s model for the real-time detection of Zanthoxylum and achieved good results in terms of both speed and precision. Tian et al. [31] proposed an improved YOLOV3 model for detecting apples at different growth stages in orchards with light fluctuations, complex backgrounds, overlapping apples, and overlapping branches and leaves. After testing, the proposed YOLOV3-dense model was found to be more effective than the original YOLOV3 model. In summary, DCNNs achieve a higher accuracy and versatility than traditional methods. High-performance computers can support their computation but cannot achieve real-time performance on embedded devices. Su et al. [32] employed a lightweight YOLOv3 model with a MobileNetV1 backbone network for tomato ripeness classification detection in a greenhouse, achieving an mAP of 97.5%. Despite its lightness, the size of the lightweight model was still 46.7 MB. Wang et al. [33] utilized an improved YOLOv3 model for the online recognition and yield estimation of tomato fruits in a PF environment, achieving a high mAP of 99.3%. However, the YOLOv3 model they employed was large-scale, rendering it difficult to apply in lightweight scenarios. This study is based on YOLOv5, with the backbone network replaced by MobileNetV3 to further reduce the model’s weight and computational load. As a result, the model is better-suited for deployment in embedded devices and harvesting robots.

In response to the demand for lightweight and high-precision tomato detection in PF environments, this study proposes the SM-YOLOv5 lightweight model for small-target tomato detection. This model addresses the limitations of current research and aims to promote the development of harvesting robots. Our research makes innovative contributions to the study of lightweight target detection algorithms and their practical applications in this field. Our contributions can be summarized as follows:

- The CSPDarknet53 backbone network was replaced by the MobileNetV3-Large lightweight network in this study. The lightweight network employed squeeze-and-excitation models and attention mechanisms to efficiently extract features in the channel-wise dimension. This replacement resulted in a reduction in the model size and a decrease in the computational demands.
- To enhance the accuracy of the lightweight model in detecting small-sized tomato fruits in images, a small object detection layer was introduced into the network architecture. This additional layer was capable of extracting more features to improve the accuracy of the detection for overlapping or small objects and obscured tomato fruits.
- These enhancements are of high importance in plant factories, where the accurate detection of small objects is crucial for effective and precise plant monitoring and management. The lightweight network can also support embedded picking robots when detecting tomato fruits, further highlighting its practical application potential.

The rest of this paper is organized as follows: Section 2 outlines the experimental environment, dataset acquisition, and processing, as well as the evaluation metrics used in this study. Section 3 describes the SM-YOLOv5 network model, the lightweight MobileNetV3-Large backbone network, and the added small-target detection layer. Section 4 provides a thorough comparison and analysis of the experimental results. Additionally, a comparison with mainstream methods and an ablation experiment were conducted. Finally, Section 5 and Section 6 provide the discussion and conclusion, respectively, including future research directions and potential improvements.

## 2. Materials and Methods

### 2.1. Data Acquisition and Preprocessing

#### 2.1.1. Image Acquisition

The data acquisition and research for this study were conducted within the Artificial Light Plant Factory Laboratory at the Henan Institute of Science and Technology (HIST), located in Xinxiang, China. The cultivar selected for the experiment was the “micro tomato”. Based on the tomato growth cycle, images were collected at multiple stages of tomato growth, commencing with tomato flowering in December 2021 and concluding with a considerable number of ripe fruits in February 2022. The collection of images was performed using a Canon 80D SLR camera, which resulted in a resolution of 6000 × 4000 pixels for 230 images. Additionally, the wide-angle camera of the iPhone 11 was used to collect 100 images with a resolution of 4032 × 3024 pixels. The compactness and convenience of the mobile phone’s wide camera enabled the collection of images from difficult angles and scenes that were not easily accessible to the DSLR camera, thus enhancing the overall diversity of the dataset.

#### 2.1.2. Dataset Annotation and Augmentation

**Figure 1:**
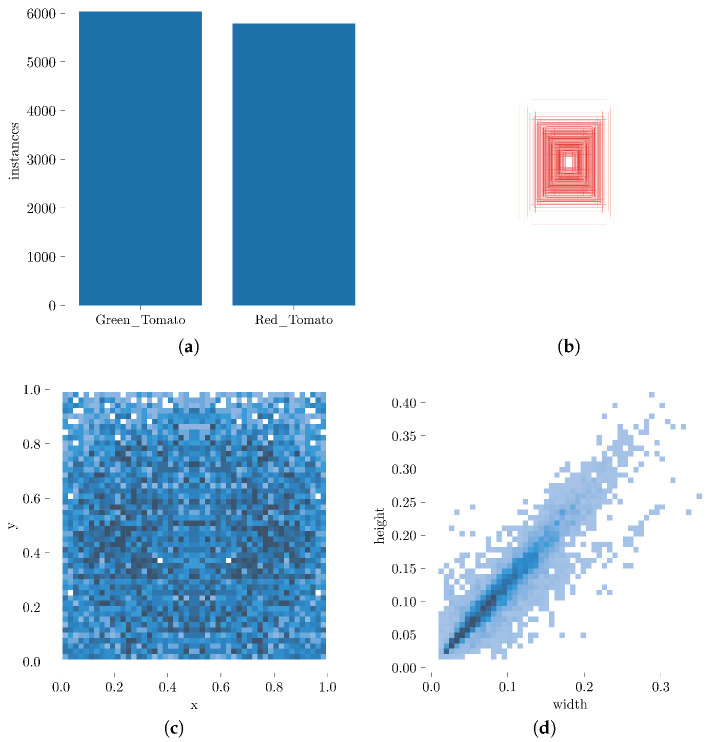
Attribute visualization results of the dataset in this study: (**a**) the number of dataset labels, (**b**) the label ratio of the dataset, (**c**) the label location of the dataset, (**d**) the label size of the data.

In order to address the deficiency of the original dataset and imbalanced samples, image processing techniques were utilized to increase the quantity of data in the initial images. Random enhancement methods were applied, including rotations of 90, 180, and 270 degrees; brightness and darkness adjustments; horizontal and vertical flipping; and the application of green and red filters. To generate tomato shapes that had not been previously encountered by the model, rotation and flipping, as well as color filtering and brightness adjustments, were used to simulate the LED lighting present in plant factories, thereby adjusting the brightness and color spectrum in accordance with the plant growth cycle. The resulting modifications to the overall scene lighting enhanced the model’s resilience and accuracy. Following random image processing, 660 images were generated, and the dataset was randomly partitioned according to a set proportion. The number of images in the dataset and sample instances is presented in Table 1 and Figure 1.

**Figure 2:**
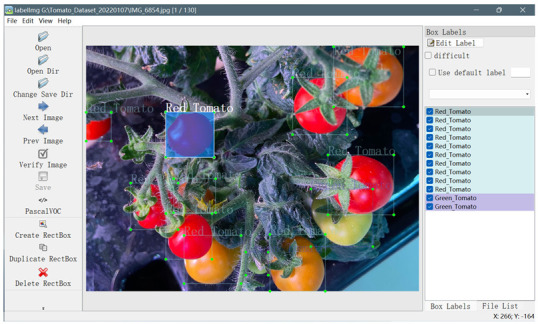
Diagram illustrating dataset annotation using LabelImg.

The collected datasets underwent processing, and LabelImg labeling software was utilized to annotate the positions of the tomatoes and their corresponding growth stages within the dataset. The resulting annotation data for each image were uniformly stored as an extensible markup language (XML) file in the visual object classes (VOC) format [34]. Figure 2 shows an example of a labeled annotation, which includes the coordinate information on the image, while the statistical outcomes of the annotation process are provided in Table 1.

Figure 1 presents the statistical outcomes visualizing the shape, distribution, and location of samples within the dataset. Figure 1a shows the distribution of the number of samples, indicating that the sample distribution was relatively uniform. Figure 1b displays the aspect ratio of the sample frame in the original image. Figure 1c demonstrates the sample center point for the entire image, with each box representing the occurrence of a sample, and the color depth reflecting the number of occurrences. The darker the color, the more frequent the occurrence. The results indicated that the distribution of sample positions in the image was relatively uniform. Figure 1d depicts the ratio of the width and height of the sample for the whole picture. Each point indicates that the sample appeared in this horizontal and vertical coordinate ratio. It can be observed that the sample concentration was near the origin, and the sample was concentrated on a ray with an oblique angle of 45°. The aspect ratio of the sample was close to a rectangle, and small-target samples dominated the dataset. Overall, the distribution and composition of the dataset were relatively uniform and reasonable, objectively reflecting the comprehensive performance of the improved small-target detection algorithm.

### 2.2. Experimental Environment

The experimental setup in this research comprised an Ubuntu 18.04 operating system and an NVIDIA GeForce RTX 3090 graphics card with 24G memory. The PyTorch 1.8.0 framework, computer unified device architecture (CUDA) version 11.1, and cuDNN version 8.3 deep neural network acceleration library were utilized for model development and training.

During the training of the model in this study, the anchor box parameters that were obtained from the K-means machine learning algorithm were set as the hyperparameters and used for training. The training, validation, and test datasets were automatically and randomly divided in a ratio of 7:2:1. The network model was initialized with pre-trained weight parameters that were obtained from training on the Common Objects in Context (COCO) dataset to accelerate the convergence of the model parameters. The cosine annealing optimization method was utilized to update the learning rate and network weight parameters, with a batch size of 32 and 600 iterations. The standard image input size was set to 640 × 640. After training the proposed SM-YOLOv5 model and a comparison model, the DCNN mainstream detection model was employed to compare and analyze the proposed method.

### 2.3. Model Evaluation Metrics

The evaluation of model performance is a crucial step in assessing a model’s detection ability and robustness. To accomplish this, a unified evaluation standard must be utilized to evaluate model performance based on the results obtained from training different models. This study employed several evaluation metrics to assess model performance, including average precision (AP), mean average precision (mAP), and floating-point operations (FLOPs). The precision, recall, and F1-score calculations are shown in Formulas (1) through (4); these metrics are commonly used in the literature [35]. A precision–recall curve (PR) could be plotted using the associated precision and recall values, and the area under the PR curve was defined as AP. The independent variable was averaged from 0 to 1, and 101 points were used to calculate the gradient integral, which was computed using Formulas (5) and (6). The mAP was determined by taking the mean value of AP for the two classifications of green and red fruits, as shown in Formula (7).

(1) $$Accuracy=\frac{TP+TN}{TP+FN+FP+TN}$$

(2) $$Precision=\frac{TP}{TP+FP}$$

(3) $$Recall=\frac{TP}{TP+FN}$$

(4) $$F1-Score=2\times \frac{Precision\times Recall}{Precision+Recall}$$

(5) $$AveragePrecision=\frac{1}{101}\sum_{r\in \{0,0.01,0.02,...,0.99\}}P_{intep}\left(r\right)$$

(6) $$P_{intep}\left(r\right)=\frac{(Precision(r)+Precision(r+0.01))\times 0.01}{2}$$

(7) $$meanAveragePrecision=\frac{\sum AveragePrecision}{sum(Class)}$$

where *TP* denotes the number of true-positive samples that were correctly classified as positive samples, *FP* denotes the number of false-positive samples that were incorrectly classified as positive samples, *FN* denotes the number of false-negative samples that were incorrectly classified as negative samples, and *TN* denotes the number of true-negative samples that were correctly classified as negative samples.

## 3. Proposed SM-YOLOv5 Model

**Figure 3:**
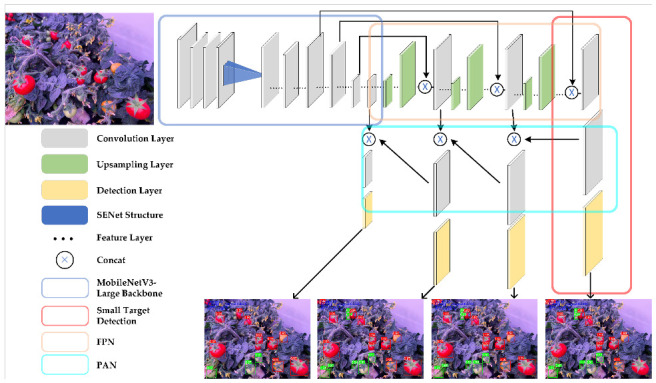
The integrated architecture of SM-YOLOv5 includes a backbone network (in blue) that was replaced with MobileNetv3-Large. The small-target detection layer added based on the original three-layer target detection model is represented by the red box. The FPN and PAN structures (in yellow and cyan boxes, respectively) were supplemented with a small object detection layer to enhance the detection of small targets.

**Figure 4:**
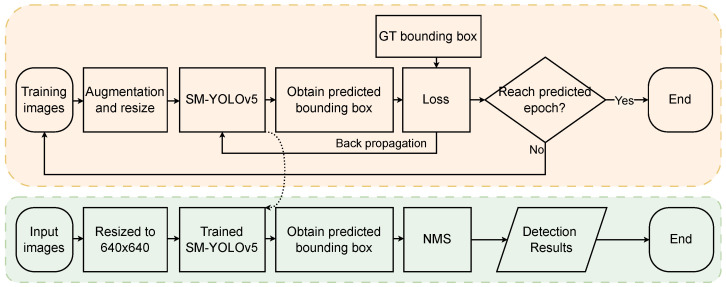
Flowchart illustrating the training and detection process of SM-YOLO, with the training phase represented by orange boxes and the detection phase represented by green boxes.

YOLOv5 is a state-of-the-art single-stage object detection algorithm that has achieved significant improvements in both accuracy and speed compared to its predecessors in the YOLO series [36]. Due to its original architecture, it can be used for the classification of 80 categories. However, in specific applications, only a few categories are typically required. In the case of tomato fruit detection, where the fruit may be small or occluded by leaves, a lightweight YOLOv5 method, namely SM-YOLOv5, is proposed in this paper. The model architecture is illustrated in Figure 3. The MobileNetV3-Large backbone was used for feature extraction, and the anchor frames, regressed by K-Means machine learning, were used in the prediction layer to train the network. A small-target scale detection layer was added to enhance the model’s ability to detect small targets. Finally, the four-layer detection content was fused for non-maximum suppression (NMS) calculation [37], and the position and classification of all tomato fruits were outputted. The model used a weight file trained on the COCO dataset [38], and the transfer learning idea was employed to accelerate the model convergence and avoid network non-convergence due to random weights. Figure 4 presents a flowchart of the training and detection process of SM-YOLOv5. Model evaluation was performed using unified evaluation standards, including AP, mAP, and FLOPs, for model comparison and evaluation. The PR curve was used to calculate the AP, and the mean value of the AP for the two classifications of green fruit and red fruit was taken as the mAP. The calculation formulas for precision, recall, and F1 score are shown in Formulas (1)–(4).

### 3.1. Lightweight MobileNetV3-Large Backbone Network

**Figure 5:**
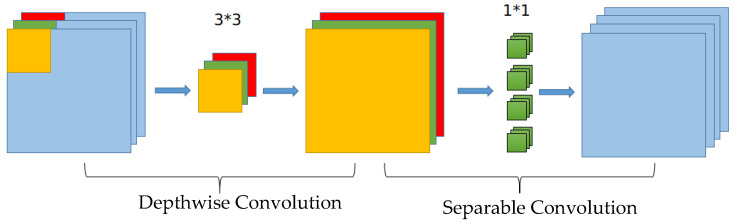
Schematic diagram of separable convolution.

The CSPDarknet53 backbone network, utilized by YOLOv5, can effectively extract image features. However, due to the high computational resources and storage space requirements, its real-time detection applicability in embedded systems is limited. To address this issue, this study proposes the SM-YOLOv5 model, which employs MobileNetV3-Large as the backbone network for a lightweight design. MobileNetV3-Large is made up of numerous bneck units. In each bneck unit, depthwise separable convolution (DWS-Conv) is employed instead of conventional convolution operations to extract features while minimizing the number of parameters and computations. As presented in Figure 5, the depthwise separable convolution is partitioned into two stages. Initially, each input channel undergoes channel-by-channel depthwise convolution (DW-Conv), and then the output undergoes pointwise convolution (PW-Conv). A depthwise weighted combination is performed, and the final feature map is output [28]. The SE module (squeeze-and-excitation) channel attention mechanism [26] is utilized in the channel separable convolution to enable the network to automatically identify the importance of each feature channel, leading to an enhanced effect. Finally, the residual network structure is utilized to alleviate the difficulty in feature transfer as the network depth increases. The detailed parameters of each layer of MobileNetV3-Large as the backbone network and the corresponding output layer are presented in Table 2 [39].

The proposed model enhances the detection of small-sized targets in real-time tomato detection by incorporating an additional layer for small-target detection, in addition to the original three layers for detecting targets of varying scales. This model fuses three layers of feature data with one layer of small-target features to achieve better performance. To improve the small-target detection accuracy, MobileNetV3-Large is used as the backbone network, and four different-sized feature layers are extracted for prediction. Table 2 presents the network architecture parameters of MobileNetV3-Large and the corresponding output layer for each of the four feature layers. The improved model balances accuracy and speed by employing 3, 640, 640 color images as input. The replaced MobileNetV3-Large backbone network extracts features of sizes $80^{2}$, $40^{2}$, and $20^{2}$ at layers 6, 13, and 15, respectively. The three-layer features correspond to small, medium, and large fields of view, which detect small, medium, and large tomato targets in the images, respectively. For detailed detection, the $160^{2}$ feature images extracted by the third layer of MobileNetV3-Large are used as the input to enable the network to detect tomato targets that are blurred or obscured in the image background, thereby improving the detection ability of panoramic images of tomatoes within the PF environment.

### 3.2. Small-Target Detection Layer

**Figure 6:**
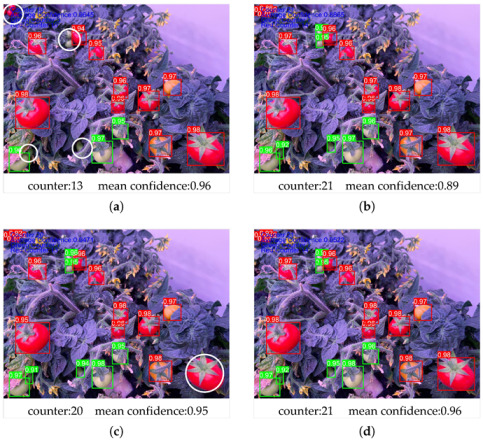
Comparison of multi-layer detection results. Detection results for (**a**) large targets, (**b**) medium targets, (**c**) small targets, and (**d**) multi-layer target fusion detection. Borders and text background colors indicate that the recognized classification was “green” or “red” fruit. White circle callouts indicate tomato fruits that were not correctly identified.

The small-target detection layer is capable of addressing the challenge of accurately detecting small, partially obscured, or blurred targets within an image. YOLOv5 has successfully implemented multi-scale target detection, thereby compensating for the shortcoming of detecting targets at a single scale [40]. The comparison of multi-scale detection in Figure 6a–c demonstrates the output results of each scale layer prediction after NMS in multi-scale detection. Figure 6a illustrates the output of the large-target detection layer, where many small-target tomato fruits were not accurately detected, but the recognized samples had a high confidence score. This could have been due to the fact that the larger tomato fruits contained more distinctive information, and the network fully extracted these features for detection. On the other hand, as shown in Figure 6c for the small-target detection layer, only one large tomato fruit was not accurately detected, and the confidence score was high. This could have been because the output of this layer involved more features in the NMS operation. The application of overlapping and merging calculations to more prediction results could enhance the confidence of the detection outcomes, as is evident for the mid-target detection presented in Figure 6b and the multi-scale fusion detection depicted in Figure 6d. In the current example, the mid-target detection layer successfully recognized all tomato fruits with a high confidence compared to the multi-scale detection layer. The multi-scale detection layer was shown to be effective in detecting targets of different sizes and improving the confidence of the detection results. The average confidence of multi-scale fusion detection was observed to be higher, and combining the detection results of the small-target layer and the large-target layer through multi-scale fusion achieved better detection results. SM-YOLOv5 was designed to use four-scale feature fusion target detection to enhance the model’s ability to detect small, partially obscured, or blurred tomato targets.

### 3.3. Trained Anchors and Transfer Learning

The anchor serves as the fundamental basis for the prediction bounding box output of a network. The prediction layer extracts multiple sets of sizes and ratios based on the anchor scale and subsequently computes the predicted classification probability and positional information of all the results. In YOLOv5, an anchor regression is performed using the K-means clustering algorithm based on the labeled sample information, after which it is assigned to the appropriate scale feature map [41]. By default, the YOLOv5 network uses the regression cluster of the COCO dataset as the highest-priority frame, and the anchors for different scale features are shown in Table 3. To improve the detection ability of deep networks for tomatoes in plant factories, their shapes were analyzed, and the optimal size of the annotation frame in the dataset was calculated. This process enhanced the accuracy and robustness of the model, and the anchor was recalculated using K-means clustering. The anchor regression clustering process used the following parameters: a cluster count of 9 or 12, twice the aspect ratio threshold, 10,000 iterations, and the determination of optimal anchors through machine learning. By utilizing this method, the accuracy and robustness of the deep network for detecting tomatoes in plant factories were improved, as evidenced by the final anchor box PR recall rate of 0.9997. The optimal parameters for the tomato anchor and tomato small-target anchor in Table 3 were obtained by performing regression clustering under the conditions of three-layer and four-layer target detection, respectively, and replacing the anchor training model with the COCO dataset. The aspect ratio of the anchor boxes obtained from the regression clustering tended to be rectangular, which aligned with the shape of the round tomato fruits present in the images.

The concept of transfer learning is employed to address issues of underfitting and convergence difficulties during model training. By utilizing network model weights pre-trained on relevant datasets, the applicability of a model to new scenarios can be improved [42]. Since the COCO dataset contains a large number of image data, the network weights trained on it could be used for the detection task presented in this paper. Thus, the proposed SM-YOLOv5 network was initialized with the COCO-trained weights, followed by the fine-tuning and training of the network. The application of pre-trained weights from a diverse range of datasets to the training of a new network can overcome the challenges associated with the slow or difficult convergence of randomly generated network weights and enhance the network’s representational capacity.

## 4. Results and Analyses

### 4.1. SM-YOLOV5 Training and Validation

**Figure 7:**
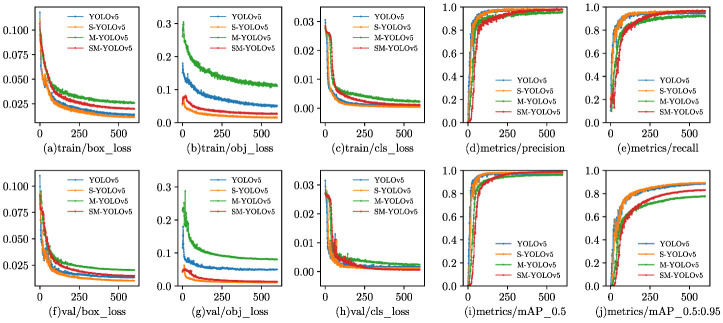
Training results of different models.

In order to provide a comprehensive comparison, all models were trained and validated using the same dataset. Figure 7 demonstrates the training process of the four models used: (1) the original YOLOv5 model; (2) YOLOv5 with a small-target detection layer, referred to as Small-YOLOv5(S-YOLOv5); (3) YOLOv5 with MobileNetv3-Large replacing the backbone network, referred to as MobileNetV3-YOLOv5 (M-YOLOv5); and (4) YOLOv5 with MobileNetv3-Large replacing the backbone network and a small-target detection layer, referred to as SM-YOLOv5. In the initial stages of all detection model training, the learning efficiency of the models was high, and the convergence speed of the training curve was fast. As the number of training epochs increased, the slope of the training curve gradually decreased and eventually stabilized. With an increase in the number of training epochs, the localization loss, confidence loss, and classification loss results changed, as shown in Figure 7a–c,f–h. It can be observed that each loss function gradually converged.

The precision and recall metrics continued to improve and converge with the increase in the number of training epochs, as depicted in Figure 7d,e. The green curve, representing the use of MobileNetV3-Large, exhibited lower performance than the original model. However, by augmenting MobileNetV3-Large with a small component, the red curve of the target detection layer ultimately aligned with the curve of the original model, indicating that the same precision and recall level as the original model were achieved.

As illustrated by the map in Figure 7i, at an intersection over union (IoU) threshold of 0.5, the model proposed in this paper attained the same level of detection accuracy as the original model while exhibiting a reduction in model scale and parameters. Conversely, as demonstrated by the map in Figure 7j, in the threshold range of 0.5 to 0.95, the proposed model’s detection performance markedly decreased, revealing a diminished ability to detect targets at higher IoU thresholds. In summary, the proposed algorithm achieved a high accuracy while reducing the model scale compared to the original algorithm. However, the model’s expression ability was observed to decrease in the high confidence interval (>0.9).

### 4.2. SM-YOLOV5 Model Testing

The proposed SM-YOLOv5 model, which is lightweight and suitable for small-target detection, was evaluated to demonstrate its effectiveness in detecting tomato fruits. The experimental results presented in Table 4 indicated that the proposed method achieved an average precision of 98.6% and 99.0% for green and red fruits, respectively, with an mAP of 98.8%. Additionally, the model required only 7.6 GFLOPs of computing power. Therefore, the proposed approach not only improved the accuracy of tomato detection but also reduced the computational burden, making it suitable for real-time detection in embedded devices. These findings provide valuable insights for the development of intelligent tomato-picking robots capable of detecting small targets.

### 4.3. Performance Comparison

To validate the proposed model’s effectiveness, the same dataset and validation set were used for model training and evaluation. The final network weights obtained from model training were used to assess the results on the same validation set. Additionally, to further verify the model’s effectiveness, we compared the proposed SM-YOLOv5 model with other mainstream DCNN target detection models. The comparison models, including SSD, YOLOv3, Faster RCNN, and YOLOv5s, were trained and tested on the same dataset. The experimental results, shown in Table 5, revealed that the SM-YOLOv5 model’s mAP reached 98.8%, which was 1.4% higher than the original YOLOv5 model. Additionally, the model’s computing power requirement was only 7.8GFLOPs, providing a noticeable computing performance advantage over other models. Thanks to the search network structure employed by MobileNetV3-Large, the detection performance reached a high level, further enhancing its computing performance advantages.

The small-target detection layer proposed in this study could effectively address the issue of reduced feature extraction resulting from the reduction in network scale. This was evidenced by the improvements in various detection performance results. The current study presents a novel target detection model that exhibited exceptional performance in terms of both detection accuracy and computational efficiency. Specifically, this model offers distinct advantages when applied to the detection of tomatoes within a PF environment.

### 4.4. Ablation Experiment

To further verify the optimization’s effectiveness, an optimization strategy ablation experiment was carried out based on the YOLOv5 model. The same training set and verification sets were used for training and verification. First, the original YOLOv5 model and each improved model were trained, and then the model performance was evaluated using the same verification dataset and method. The test results are shown in Table 6. The test results showed that using the more streamlined MobileNetV3-Large to replace the CSPDarknet53 backbone network could effectively reduce the network size from the required computing power of 15.8 GFLOPs to 4.7 GFLOPs, theoretically increasing the model prediction speed threefold. The model’s accuracy was reduced due to the small parameter scale of the proposed model. Adding a small-target detection layer to the original model was proposed to improve the model results. The experimental results showed that the detection effect of the model could be increased. By adding a small-target detection layer to M-YOLOv5 to form the SM-YOLOv5 model, the computing power was increased to 7.6 GFLOPs; however, this was only half that of the original model. In theory, the detection speed could be doubled, and the mAP also reached 98.8%; all aspects of the performance matched the level of the original model and improved upon it.

**Figure 8:**
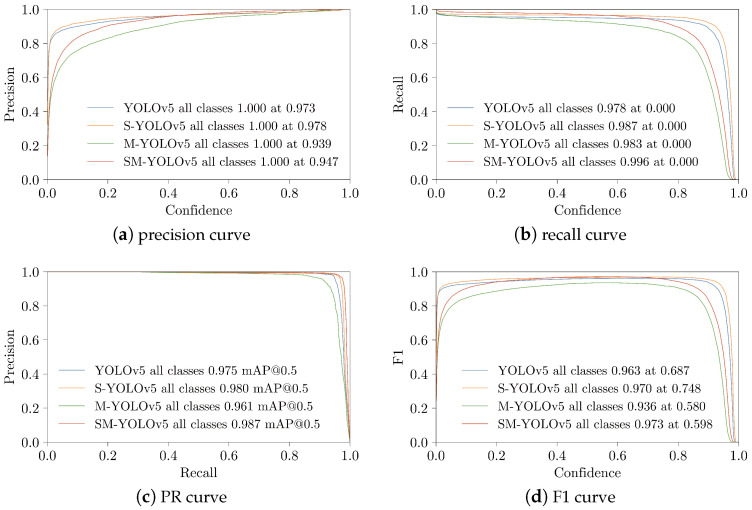
Visualization of the results from ablation experiments conducted using YOLOv5, S-YOLOv5, M-YOLOv5, and SM-YOLOv5 methods.

The training results presented in Figure 8 illustrate the performance evaluation based on the ablation experiment. The curve intuitively reflects the change in each performance index with confidence. Specifically, in the PR curve in Figure 8a and Formula (2), the accuracy reflects the proportion of predicted positive samples corresponding to the ground truth (GT) samples at a particular confidence level, indicating the performance of the model verification and false-detection rate. Lower confidence levels may have yielded some low-confidence false-detection results corresponding to GT samples, which led to a higher accuracy. However, as the confidence level increased, the false-detections with a low confidence and the results with a low confidence were filtered and excluded, resulting in reduced accuracy. This trend was intuitively reflected in the curve, which shows that the model’s accuracy gradually decreased with the increase in the confidence level.

Furthermore, the comparison between the proposed and original models in Figure 8a shows that the curve of the original model rose earlier, whereas the proposed model’s curve inclined more slowly in the low-confidence interval. However, the proposed model surpassed the original model later, and it reached the maximum accuracy of 1 first.

In the PR curve and Formula (3) shown in Figure 8b, the recall rate reflects the proportion of predicted positive sample results corresponding to ground truth samples and all positive sample results predicted under a specific confidence level. It reflects the model’s performance in recalling actual positive samples and identifying missed checks. At low confidence levels, the model predicted more samples, and the proportion corresponding to ground truth was also higher, which was reflected in the recall rate. However, as the confidence level increased, the predictions were gradually filtered out, and the recall rate decreased until it reached a minimum value of 0.

Figure 8b reveals that the proposed model was more sensitive in the high-confidence interval and declined earlier compared to the original model. This may have been due to the proposed model’s utilization of a small-scale backbone network, resulting in less-sufficient feature extraction than the original model. The proposed model dropped sharply after the confidence level reached 0.8, which indicated a decrease in the model’s expression ability. In contrast, the original model dropped sharply after the confidence level reached 0.9. When combined with the precision curve and application procedure, the recall rate was significantly reduced. Nonetheless, confidence intervals for higher performance could still be obtained.

The PR curve and Formulas (5)–(7) depicted in Figure 8c associate the confidence value with two values, and the area under the curve represents the model’s mAP value. The formula describes the discontinuous gradient integration method utilized during the model training, which comprehensively reflects the model’s performance. The figure shows that the performance of the proposed model was relatively close to but improved upon that of the original model, meeting the requirements for application.

In Figure 8d and Formula (4), the F1 score is the harmonic mean of precision and recall, which provides a comprehensive performance index for the model and its maximum performance point. The proposed model achieved the same maximum value as the original model without significant performance degradation.

Comparing the curves in Figure 8a,b, it is observed that the accuracy of the proposed model was more sensitive at a low confidence and recall and at a high confidence, which was also reflected in the F1-score curve. The F1-score curves were slightly lower than the original model at low-confidence intervals (less than 0.2) and high-confidence intervals (higher than 0.8). However, this did not affect the proposed model’s high speed and accurate detection in the best confidence interval.

## 5. Discussion

To address the problem of the difficulty of using general deep learning target detection technology to detect the different growth states of tomato fruits while ensuring a light weight and high precision in the environment of plant factories, this paper proposed a YOLOv5 tomato fruit growth state detection model using the MobileNetV3-Large backbone network.

The method was based on the YOLOv5 network model and used MobileNetV3-Large to replace the CSPDarknet53 backbone network for efficient feature extraction. The search structure used by MobileNetV3-Large can efficiently extract enough features on small-scale networks. For tomato fruits, the image tends to be rectangular in proportion; therefore, the K-Means clustering algorithm was used for regression in the tomato dataset to obtain the best anchors and participate in network training as a hyperparameter. In order to address the insufficiencies arising from the utilization of the MobileNetV3-large backbone network, we proposed the incorporation of a small-target detection layer to improve the three-layer multi-scale detection structure of the original model. This augmentation is particularly well-suited to scenarios in which the target object, in this case tomato fruit, is comparatively small within the context of plant factories; by integrating a smaller backbone network with an additional small-target detection layer, our network was capable of predicting all relevant features with minimal computational overhead, requiring only 7.6 GFLOPs. Furthermore, our approach achieved an mAP score of 98.7% and had a compact model file size of only 6.3 MB.

The approach outlined in this paper achieved the accurate and efficient detection of tomato fruits in testing scenarios, with a performance on par with and improved compared to that of the original YOLOv5 model. The subsequent steps involve implementing the model for detection in embedded systems and robots, refining the network model to enable the real-time detection of tomato fruit growth status in tomato-picking robots, and collaborating with multi-eye 3D cameras to precisely locate the tomato fruits within the robot’s workspace. By achieving accurate coordinate positioning, the aim is to enable high-precision, automated picking by robots.

## 6. Conclusions

In the present study, we proposed a novel algorithm, SM-YOLOv5, for the detection of tomato fruits in a PF laboratory environment. Our algorithm was specifically designed to satisfy the lightweight requirement of picking robots and the high-precision demands of control systems employed in plant factories. Based on the experimental research and result analysis conducted in this study, the main findings were as follows:

1. Lightweight: The proposed model backbone was replaced with the MobileNetV3-Large network, which is a lightweight architecture that reduced the model’s FLOPs to 7.6 GFLOPs and its size to 6.3 MB.
2. Small-target detection: The additional detection layer resulted in the improved performance of the proposed algorithm in detecting tomatoes that were obscured, overlapping, or small in size.
3. Accuracy: The proposed model was modified to reduce its scale by replacing the backbone with a lightweight alternative. To ensure accurate detection while maintaining the model’s lightweight characteristic, a small detection layer was integrated into its architecture. This operational enhancement resulted in a significant improvement in accuracy, with the test set achieving a score of 98.8%.

In contrast to conventional detection algorithms, SM-YOLOv5 exhibited robustness in accurately detecting tomato fruits while effectively identifying targets that were far-off, partially obscured, and overlapping in PF environments. Moreover, the lightweight characteristic of the model proposed in this paper provides significant advantages for the design of picking robots and control systems.

## Figures and Tables

**Table 1:** Number of tomato images and sample instances in the constructed dataset.

Set	Number of Images	Number of Green Tomato Samples	Number of Red Tomato Samples
Training	462	4183	4104
Validation	132	1240	1160
Testing	66	607	522
Total	660	6030	5786

**Table 2:** Parameters of MobileNetV3-Large network architecture and corresponding prediction layers.

Layer	Input Size	Kernel Size	Expand	#out ^1^	SE ^2^	NL ^3^	s ^6^	Detection Layer
1	320, 320, 8	3 × 3	16	16		RE ^4^	1	
2	320, 320, 8	3 × 3	64	24		RE	2	
3	160, 160, 16	3 × 3	72	24		RE	1	Detection1 ^5^
4	160, 160, 16	5 × 5	72	40	✓ ^7^	RE	2	
5	80, 80, 24	5 × 5	120	40	✓	RE	1	
6	80, 80, 24	5 × 5	120	40	✓	RE	1	Detection2 ^5^
7	80, 80, 24	3 × 3	240	80		HS ^4^	2	
8	40, 40, 40	3 × 3	200	80		HS	1	
9	40, 40, 40	3 × 3	184	80		HS	1	
10	40, 40, 40	3 × 3	184	80		HS	1	
11	40, 40, 40	3 × 3	480	112	✓	HS	1	
12	40, 40, 56	3 × 3	672	112	✓	HS	1	
13	40, 40, 56	5 × 5	672	160	✓	HS	2	Detection3 ^5^
14	40, 40, 80	5 × 5	960	160	✓	HS	1	
15	20, 20, 40	5 × 5	960	160	✓	HS	1	Detection4 ^5^

^1^ The term “out” indicates the dimension size of the output. ^2^ “SE” indicates whether the layer uses the squeeze and excite structure. ^3^ “NL” indicates the type of nonlinear function. ^4^ “HS” indicates the use of the h-swish nonlinear function, and “RE” indicates the use of the ReLU nonlinear function. ^5^ “Detection 2,3,4” are the three-layer detection layers of the original YOLOv5 model, and “Detection 1” is the small-target detection layer added in this paper. ^6^ “s” indicates the step size. ^7^ “✓” indicates that SE is used in the layer.

**Table 3:** A set of anchor values for COCO, tomato, and tomato small-target detection.

Downsampling	COCO Anchor	Tomato Anchor	Our Anchor
2×			
4×			18 × 17, 22 × 23, 26 × 25
8×	10 × 13, 16 × 30, 33 × 23	19 × 19, 30 × 29, 40 × 38	34 × 34, 52 × 52, 45 × 44
16×	30 × 61, 62 × 45, 59 × 119	51 × 49, 62 × 63, 76 × 73	57 × 53, 65 × 66, 81 × 76
32×	116 × 90, 156 × 198, 373 × 326	89 × 85, 108 × 106, 132 × 130	93 × 91, 110 × 108, 134 × 133

**Table 4:** Detection results for tomato fruit using SM-YOLO model.

Tomato Fruit Color	Precision (%)	Recall (%)	AP (%)	mAP (%)
Green	98.0	96.2	98.6	98.8
Red	98.5	96.8	99.0	

All precision and recall values were obtained at a confidence level of 0.59, which corresponded to the peak point in the F1 curves of both categories.

**Table 5:** Comparison of training and validation results for YOLOv5, SSD, YOLOv3, Faster RCNN, and SM-YOLOv5.

Network	Backbone	Number of Detection Layers	mAP (%)	GFLOPs	Weight Size (MB)
YOLOv5	CSPDarknet53	3	97.4	15.8	14.9
SSD	VGG16	6	90.7	30.5	182.0
YOLOv3	Darknet53	3	97.5	154.9	470.2
Faster RCNN	VGG16	Regional proposal	81.2	63.9	522.0
SM-YOLOv5	MobileNetV3-Large	4	98.8	7.6	6.3

**Table 6:** Training and validation result comparison between YOLOv5, SSD, YOLOv3, Faster RCNN, and SM-YOLOv5 models.

Network	Small-Target Detection Layer	Improved Backbone	Detection Layer	Precision (%)	Recall (%)	mAP (%)	GFLOPs	Weight Size (MB)
YOLOv5			3	97.9	94.8	97.4	15.8	14.9
S-YOLOv5	✓ ^1^		3 + 1	98.0	96.2	98.0	23.4	14.9
M-YOLOv5		✓	3	97.9	95.4	98.3	4.7	6.33
SM-YOLOv5	✓	✓	3 + 1	97.8	96.7	98.8	7.6	6.33

Since the values of precision and recall were dependent on the confidence threshold, the values reported in the table correspond to the confidence threshold at which the maximum value of the F1 score was achieved. ^1^ “✓” indicates that the improvement is used in the model.

## Data Availability

The data presented in this study are available from the first author upon request.

## Abbreviations

The following abbreviations are used in this manuscript:

CHT	Circular Hough transform
CNNs	Convolutional neural networks
COCO	Common Objects in Context
CUDA	Computer unified device architecture
DCNNs	Deep convolutional neural networks
DW-Conv	Depthwise convolution
DWS-Conv	Depthwise separable convolution
FLOPs	Floating-point operations
FNCC	Fast normalized cross-correlation function
GT	Ground truth
HOG	Histogram of oriented gradient
IoU	Intersection over union
M-YOLOv5	Mobilenet-YOLOv5
NMS	Non-maximum suppression
PF	Plant factory
PW-Conv	Pointwise convolution
RCNN	Regional convolutional neural network
SIFT	Scale-invariant feature transform
S-YOLOv5	Small-YOLOv5
SM-YOLOv5	Small-Mobilenet-YOLOv5
SSD	Single-shot MultiBox detector
SVM	Support vector machine
VOC	Visual object classes
XML	Extensible markup language
YOLO	You Only Look Once
